# Biceps Tendon Lengthening Surgery for Failed Serial Casting Patients With Elbow Flexion Contractures Following Brachial Plexus Birth Injury

**Published:** 2016-08-30

**Authors:** Rahul K. Nath, Chandra Somasundaram

**Affiliations:** Texas Nerve and Paralysis Institute, Fannin St, Houston, TX 77030

**Keywords:** elbow flexion contracture, limb length discrepancy, serial casting, biceps tendon lengthening, obstetric brachial plexus injury

## Abstract

**Objective:** Assessment of surgical outcomes of biceps tendon lengthening (BTL) surgery in obstetric brachial plexus injury (OBPI) patients with elbow flexion contractures, who had unsuccessful serial casting. **Background:** Serial casting and splinting have been shown to be effective in correcting elbow flexion contractures in OBPI. However, the possibilities of radial head dislocations and other complications have been reported in serial casting and splinting. Literature indicates surgical intervention when such nonoperative techniques and range-of-motion exercises fail. Here, we demonstrated a significant reduction of the contractures of the affected elbow and improvement in arm length to more normal after BTL in these patients, who had unsuccessful serial casting. **Methods and Patients:** Ten OBPI patients (6 girls and 4 boys) with an average age of 11.2 years (4-17.7 years) had BTL surgery after unsuccessful serial casting. **Results:** Mean elbow flexion contracture was 40° before and 37° (average) after serial casting. Mean elbow flexion contracture was reduced to 8° (0°-20°) post-BTL surgical procedure with an average follow-up of 11 months. This was 75% improvement and statistically significant (*P* < .001) when compared to 7% insignificant (*P* = .08) improvement after serial casting. **Conclusion:** These OBPI patients in our study had 75% significant reduction in elbow flexion contractures and achieved an improved and more normal length of the affected arm after the BTL surgery when compared to only 7% insignificant reduction and no improvement in arm length after serial casting.

Patients with permanent obstetric brachial plexus injury (OBPI) develop persistent biceps contracture and loss of extension of the elbow if they do not fully recover. The flexed elbow posture not only limits upper extremity functions but also may cause pain. These OBPI patients with elbow flexion contractures (EFCs) result in shortening of the affected arm and limiting functions of the forearm and hand.^1^^-^3 An EFC further worsens the limb length and overall upper extremity functions of the affected arm. The reported prevalence of EFC in OBPI is ranging widely between 4.6% and 89.5%.^1^^,^^4^^-^6

Serial casting and splinting have been shown to be effective in correcting EFCs in OBPI by Ho et al.^7^ However, they have also reported the possibilities of radial head dislocations and other complications in serial casting and splinting. Literature indicates surgical intervention when such nonoperative techniques and range-of-motion exercises fail.

Occupational therapy and long-term night splints usually manage OBPI with EFC limited to 15° to 20°. The deficit can be severe (30°-80°) in some OBPI patients and this is treated surgically.^1^^,^^7^^-^11 Standard surgical methods of correction are soft tissue release at the elbow joint in conjunction with a lengthening of the biceps, brachialis, and flexor-pronator mass.^12^^-^18

Here, we report the successful outcome of biceps tendon lengthening (BTL) on 10 OBPI patients, who were resistant to serial casting and had EFC and significantly shorter arm when compared with the unaffected arm after serial casting.

## PATIENTS AND METHODS

Ten OBPI children (4 boys and 6 girls) with an average age of 11. 2 years (4-17.7 years), who had serial casting and still with 30° to 50° EFC underwent BTL surgery^12^ with us. All 10 of these OBPI patients (100%) in this study had failed serial casting. Average follow-up time was 11 months (6-16 months). This was a retrospective study of patient charts, which exempted it from the need for the institutional review board approval in the United States. Patients were treated ethically in compliance with the Helsinki declaration. Documented informed consent was obtained for all patients.

### Statistical analysis

Paired Student *t* tests were conducted using Microsoft Excel 2003 with the Analyze-It plug-in (Redmond, WA; and Leeds, UK) to determine:

1. If there was a statistically significant difference between serial casting and BTL surgery in reducing EFCs and improving arm length in OBPI patients.
2. If there was a statistically significant difference in reducing EFCs and improving arm length after serial casting in these patients.
3. If there was a statistically significant improvement in these patients after BTL surgery. The *P* values were 2-tailed and considered significant if less than or equal to .05.

## RESULTS

All 10 patients in this study documented to have had shoulder dystocia. Two had Horner syndrome. Seven of them were delivered (documented) using either vacuum or forceps or both. One was documented to have finger movement at birth, and no finger movement was documented for other patients at birth. Of 10 patients in our study group, 5 patients had C5-C7 and one with C5-C6 upper plexus injury, one had C5-C8, and 3 had total brachial plexus injury (Table 1). The mean severity of the contractures in C5-C7 patients as well as in total plexus injury patients was 40° (30°-50°) (Table 1). This indicates that the extent of the contractures is not significantly associated with the severity of the injury.

**Figure 1:**
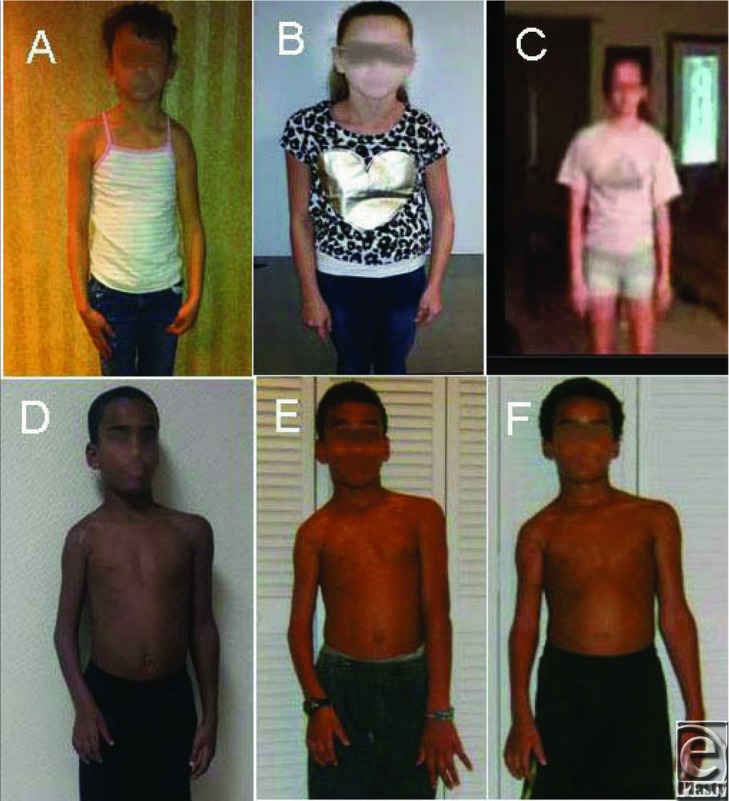
The outcome of serial casting versus BTL in a 12-year-old OBPI girl (upper panel) and a 10-year-old OBPI boy (lower panel). Pictures A and D show before serial casting. Pictures B and E show after serial casting but before BTL surgery. Pictures C and F show after BTL surgery. BTL indicates biceps tendon lengthening; OBPI, obstetric brachial plexus injury.

Mean flexion contracture was 40° (30°-50°) before serial casting in our study group. There was persistent EFC (30°-50°; mean 37°) with only 7% reduction after serial casting (Table 1). Therefore, these patients underwent BTL surgery with us. Elbow flexion contracture reduced to 0° to 20° post-BTL surgical procedure with an average follow-up of 11 (6 -16) months (Table 1). We have demonstrated that the BTL corrected the EFC by reducing an average of 30° and improved the arm length and upper extremity functions (Table 1 and Fig 1). This was 75% improvement and statistically significant (*P* < .001) when compared with 7% insignificant (*P* = .08) improvement after serial casting.

The mean reduction of the EFCs was 30° in patients with C5-C7 and in patients with total plexus injury after BTL surgery (Table 1). Our results show that the outcome of this surgery was not significantly different in terms of the severity of the injury in this group of patients.

## DISCUSSION

We have previously reported that neurolysis with tetanic stimulation of median, radial, and ulnar nerves can have a significant impact on the elbow joint contractures caused by OBPI.^12^ Here, we have demonstrated that combined with BTL surgery and contracture release, we were able to achieve significant improvements (*P* < .001; 75%) in arm length and function in 10 OBPI patients, who had unsuccessful serial casting (*P* = .08; 7%).

The outcome of the surgery and the severity of the contractures in our patients with C5-C7 and total brachial plexus injury were not significantly different in this study group.

We demonstrated here that the BTL surgical procedure corrected the EFCs, at about an average of 30°, and improved the arm length significantly when compared with serial casting, and, therefore, the upper extremity functions.

## CONCLUSION

These OBPI patients in our study had not only 75% and a significant reduction in EFCs and they also achieved an improved and more normal length of the affected arm after the BTL surgery when compared to only 7% insignificant improvement and no change in arm length after serial casting.

## Figures and Tables

**Table 1:** Statistically significant outcome of BTL versus serial casting in OBPI*

Patient	Age (years at surgery)	Nerve involved	Elbow flexion contracture pre-serial casting	Elbow flexion contracture post-serial casting	Elbow flexion contracture post-BTL	Follow-up months
Female	11.9	Total	30	30	0	6
Female	12.8	C5-C7	40	30	0	8
Female	15.4	C5-C7	50	40	10	15
Male	9.4	Total	50	50	10	9
Female	17.7	Total	30	30	0	9
Male	8.6	Total	50	50	20	9
Male	12.5	C5-C7	50	50	30	15
Male	13	C5-C7	30	30	0	16
Female	4	C5-C7	40	30	0	9
Female	8.7	C5-C8	30	30	10	11
Mean	11		40°	37°	8°	11
SD			9.4	9.5	10.3	
*P* value				.08	.001	

*BTL indicates biceps tendon lengthening; OBPI, obstetric brachial plexus injury.
